# Community service nurses’ experiences regarding their clinical competence

**DOI:** 10.4102/hsag.v24i0.1284

**Published:** 2019-10-21

**Authors:** Kholofelo L. Matlhaba, Abel J. Pienaar, Leepile A. Sehularo

**Affiliations:** 1 Department of Health Studies, University of South Africa, Pretoria, South Africa; 2 School of Health Sciences, Sefako Makgatho Health Sciences University, Pretoria, South Affrica; 3 School of Nursing Science, North-West University, Mahikeng, South Africa

**Keywords:** clinical competence, community service, community service nurse, experiences, placement

## Abstract

**Background:**

In South Africa, it is mandatory for nurses who have qualified as a nurse (general, psychiatric and community) and midwifery, leading to registration in Government Gazette Notice No. R425 of 22 February 1985, to perform 12 months’ compulsory community service after completion of training at a College of Nursing. Community service affords new graduate nurses the opportunity to improve their clinical skills and knowledge while nurturing professional behavioural patterns and critical thinking consistent with the profession.

**Aim:**

To explore and describe the experiences of community service nurses (CSNs) regarding clinical competence during their placement in three selected hospitals.

**Setting:**

The study setting was North West Province (NWP), South Africa.

**Method:**

This study followed a qualitative, exploratory, descriptive and contextual research design. A cluster sampling technique was used and 17 CSNs participated in the study. Three focus group discussions framed by semi-structured questions were conducted with five to six participants per group. All discussions were recorded using a digital voice recorder and transcribed. Data were analysed using Pienaar’s four steps of qualitative thematic analysis.

**Results:**

Four themes emerged from this study: facilitative experiences, defacilitative experiences, challenges confronted during placement and suggestions to improve clinical competence.

**Conclusion:**

Clinical competence of CSNs could be improved if all the stakeholders, including professional nurses and CSNs themselves, hospital management and the regulatory body, the South African Nursing Council, collaborate. More importantly, this study’s results were used to develop a clinical competence evaluation tool in the NWP, South Africa.

## Introduction

In many countries around the world, new nurses are associated with under-preparedness and a low level of clinical competence, which lead to their inability to provide quality nursing care (Duchscher 2009). This is a concern around the world where either the nursing education institutions (NEIs) or the government resorts to introducing measures that seek to improve the competencies of newly qualified nurses (Duchscher 2009). According to Andre and Barnes (2010), many hospitals offer a 12-month non-compulsory graduate nursing programme designed to support and build the confidence and competence of graduate nurses as they develop professionally. This is perceived to assist and support graduate nurses in their transition into the working environment (Andre & Barnes 2010). In the United States, The Institute of Medicine (IOM) (2011) has determined that nursing graduates need to enhance competencies in the basic areas of nursing care (IOM 2011). Institute of Medicine suggests that to ensure that its members are well prepared, the profession should institute residency training for nurses (IOM 2011). Furthermore, according to Theisen and Sandau (2013), many health organisations use orientation programmes or nurse residency programmes to ensure that new graduates are both confident and competent.

## Background

In the Republic of South Africa (RSA), it is mandatory for nurses to complete a 12-month community service programme after the successful completion of a 4-year nursing degree or nursing diploma education (R425) before they can be registered as professional nurses (general, psychiatric or community) and midwifery (South African Nursing Council [SANC] 2005). This requirement has been published and further details about community service for this category of nurses at the health care facilities have been enumerated in the Government Gazette Notice No. 765 of 24 August 2007. Community service for nurses in the RSA was implemented in 2008 (Govender, Brysiewick & Bhengu 2017). According to the Department of Health, the process of community service affords young South African health professionals the opportunity to develop skills, and acquire knowledge, behaviour patterns and critical thinking that will assist them in their professional development (South Africa. Department of Health 2006).

Competence is defined as the constellation of abilities, including knowledge, skills and attitudes across multiple domains of performance in a certain context (Riddle, Beker & Sapp 2016). Kubin and Fogg (2010) describe competence as the ability to perform tasks according to defined expectations. According to Hansen-Salie and Martin (2014), clinical competence is the ability of newly qualified nurses to integrate theory into practice and to render quality patient care. In this study, clinical competence refers to the abilities of the community service nurses (CSNs) to work competently in providing quality nursing care to the patient(s) under their care during community service period. In the North West Province (NWP) of South Africa, like other provinces, CSNs perform their 12-month community service in allocated health care facilities such as clinics and hospitals under the supervision of experienced professional nurses in order to obtain and improve their clinical experience (SANC 2007). As newly qualified nurses, CSNs require the supervision and support of experienced professional nurses to enhance their clinical competence to ensure seamless transitioning into the working environment as professional nurses after completion of the mandatory community service (Makhakhe 2010).

Community service nurses’ preparedness and readiness to enter the working environment has been questioned for various reasons. This is evident in several studies conducted and reported on the clinical competence of CSNs since the implementation of community service for nurses in 2008 in the RSA. A qualitative study conducted by Shezi (2014) in the KwaZulu-Natal Province revealed that not all CSNs were fully competent and independent to allow them to practice autonomously during their community service. Shezi (2014) further reports that the competence of CSNs developed in the period of community service was significantly influenced by the clinical supervision provided by experienced registered nurses who assisted with continued development of skills in clinical practice (Shezi 2014). In a comparison study conducted in the Western Cape by Snell and Daniels (2014), professional nurses perceived the CSNs who had completed a diploma nursing programme to be more competent than nurses with university degrees. In addition, the study conducted by Khunou (2016) in the NWP revealed that nursing service managers perceived CSNs as lacking in practical skills, professional responsibility and these CSNs were unable to exhibit basic practical skills. The same sentiments were shared by Makua (2016) whose study reported that operational nurse managers perceived newly qualified nurses as lacking in clinical competence during community service (Makua 2016).

In a qualitative study conducted by Netshisaulu and Maputle (2018) in the Limpopo Province, it was identified that new graduate midwives placed for community service lacked a sense of independence and commitment to patient care and could not perform delegated duties with respect to ward coverage as per the expectations of experienced midwives. This lack of independence and commitment was seen to have an impact on the increased workload and frustration of the experienced midwives supervising CSNs (Netshisaulu & Maputle 2018). Therefore, experiences of CSNs in the NWP regarding their clinical competence during placement need to be investigated. The findings and recommendations of this study could assist to improve the experiences of CSNs regarding their clinical competence during placement in the NWP, South Africa.

### Theoretical perspective

This study followed the point of competence development stages by theorist Patricia Benner (2001). Clinical competence forms the basis of nursing practice. In its nursing education and training standards under the provision of *Nursing Act No 33, 2005*, the SANC emphasises the importance of clinical competence among nurses. According to Benner’s Novice to Expert Theory (Benner 2001), a person passes through five levels of proficiency, with the third level being competence, which the SANC defines as a combination of knowledge, psychomotor, communication and decision-making skills that enable an individual to perform a specific task at a level of proficiency. It is believed that community service programme encourages competence development for nurses (SANC 2007).

## Methods

### Research setting

Three selected hospitals formed the research setting for this study in the NWP, South Africa. One was a district level 1 hospital and the other two were regional level 2 hospitals. These hospitals were selected based on the availability of CSNs for focus group discussions (FGDs).

### Research design

This study utilised a qualitative, exploratory, descriptive and contextual qualitative research design (Creswell 2014) aimed at gaining an in-depth understanding of CSNs’ experiences during community service placement.

### Sampling method

A cluster sampling method was used to select participants for this study. Cluster sampling technique is used when the elements of a population are spread over a wide geographical area (Creswell & Creswell 2018). According to De Vos et al. (2011), cluster sampling is employed when economic considerations and cluster criteria are significant for the study. In this study, CSNs are placed across all the four regions of the NWP, which made it impossible for the researcher to extend the study across the entire population (Babbie 2015). Therefore, three hospitals were selected with one FGD each. Inclusion criterion for this study was the CSNs who were performing their community service at the selected hospitals. Community service nurses who had completed their community service but were awaiting registration by SANC were excluded from this study.

### Instrumentation for data collection

The FGDs were framed by semi-structured questions to reach an informed understanding of the participants’ lived experiences and to increase the credibility of the findings (Creswell & Creswell 2018). The interviews were audio-recorded. The interview guide was self-developed, pretested and narrowed to two central questions with possible probes. The questions were as follows:

What are your experiences regarding clinical competence during your placement as a CSN?

What suggestions do you have for improving clinical competence of CSNs during placement?

### Data collection process

Data were collected through FGDs. Focus group discussion is a research technique used to collect data through group interaction (De Vos et al. 2011). Data were collected between September and November 2018. Three FGDs with 17 CSNs (two groups with six and one group of five) informed this study. The first author conducted all the FGDs. Data were collected in each FGD until saturation was reached (Creswell & Creswell 2018). These FDGs were conducted during working hours when participants were on duty as arranged with the nursing service managers of the selected hospitals. The researcher, being a former nurse lecturer at one of the NEIs in the province, ensured that all personal knowledge and theoretical knowledge were bracketed so that full attention could be given to investigating the clinical competencies and challenges experienced by CSNs (De Vos et al. 2011). This was achieved by attempting to withhold all prior knowledge and past experiences, which would contaminate the studied phenomenon (Polit & Beck 2012).

### Data analysis

All FDGs were recorded and transcribed. Data from the transcripts were analysed independently by the first and third authors per Pienaar’s four steps of qualitative thematic analysis (Pienaar 2017). The steps are set out as follows:

**Step 1:** Data collection and analysis occur simultaneously. Basic concepts are derived from the spoken words. The researcher used a recorder to record spoken words and take field notes.

**Step 2:** Group similar concepts together. Constantly, as the concepts emerged, the researcher separated and categorised related concepts together.

**Step 3:** The researcher intuitively deduced, converged and identified new concepts, themes or clusters (insights).

**Step 4:** Build the storyline or pattern to form a process and a generic framework for an African unique context. A discussion was held by the first and the third author to verify the final themes and reach consensus on the veracity and authenticity of the themes and patterns.

### Measures of trustworthiness

Trustworthiness of the results was ensured by following the criteria suggested by Creswell and Creswell (2018). Credibility was ensured because the FGDs were conducted by the researcher herself, who is experienced in conducting FGDs. Credibility was also ensured by checking the reliability of coding with a qualified researcher who analysed the data and checked for patterns (Creswell & Creswell 2018). Furthermore, dependability was ensured by selecting a solid description of the research methods and the appropriateness of the methodological applications. A digital recorder was used and an independent transcriber transcribed raw data and the researcher validated the transcripts. To ensure transferability, the researcher generated thick descriptions of the setting, process of data collection, analysis methods and results. Existing literature was used to confirm or dismiss the perceptions, and a dense description of the results was provided to enhance comparison with other similar settings. Lastly, conformability was ensured by an inquiry audit, in which the identified themes and sub-themes were scrutinised by an experienced independent coder.

### Ethical considerations

The scientific committee of the School of Nursing Science (SONS), Faculty of Agriculture, Science and Technology (FAST) Health Science Ethics Committee (HSEC) of the North-West University first approved this study. The North West Department of Health and the three selected hospitals in the NWP, South Africa, also granted ethical clearance for this study. Informed consent was obtained from the CSNs after a thorough explanation of what was expected of them during their voluntary participation in the study. Community service nurses were offered the choice to participate in this study, and they were informed that they could withdraw from participating without being penalised. The study posed minimal risk as CSNs may experience discomfort or emotions when talking about their experiences. The identity of all CSNs was safeguarded by using card numbers as identity codes. Discussion of results was done in such a way that CSNs cannot be identified through of the use of identity codes. Fairness was ensured during participation as all participants were given an opportunity to express their experiences without bias or coercion from the researcher. The study would be of indirect benefit to participants, as their contribution to the developed tool will be of benefit to the future CSNs in the province.

#### Demarcation and description of the sample

Three FGDs were conducted with CSNs at the three selected hospitals in the NWP. All of these CSNs were allocated to the three selected hospitals for their community service. Two FGDs consisted of six CSNs and one FGD consisted of five CSNs. Table 1 reflects the hospitals and its classification involved in the study, the number of CSNs per hospital, age, gender, race and the qualification obtained.

**TABLE 1:** Description of the sample for focus group discussions.

Demographic data	Hospital A (District Level 1)	Hospital B (Regional Level 2)	Hospital C (Regional Level 2)
Age ranges	22 – 29 = 3	26 – 29 = 2	23 – 29 = 2
	30 – 39 = 1	30 – 39 = 2	30 – 39 = 3
	40 – 49 = 2	40 – 49 = 1	40 – 49 = 1
Gender	4 females and 2 males	3 females and 2 males	4 females and 2 male
Race	4 black people and 2 white people	5 black people	4 black people and 2 white people
Qualification obtained	4 degrees and 2 diplomas	3 diplomas and 2 degrees	4 diplomas and 2 degrees
Province of training	5 North West and 1 KwaZulu-Natal	5 North West	6 North West

## Presentation and discussion of results

Table 2 provides a summary of the themes and sub-themes, and discussions with literature control follows:

**TABLE 2:** Themes and sub-themes.

Themes	Sub-themes
1. Facilitative experiences of CSNs	1.1. Improved clinical competence 1.2. Effective teamwork among staff members 1.3. Supportive nursing staff and other health professionals 1.4. Constructive orientation and supervision
2. Defacilitative experiences of CSNs	2.1. Unrealistic expectations 2.2. Incompetent to perform basic nursing procedures 2.3. Undesirable attitudes from some permanently employed staff 2.4. Lack of interest in specific ward/department (maternity)
3. Challenges confronted during placement	3.1. Shortage of human and material resources 3.2. Unavailability of a job description or scope of practice 3.3. Inconsistent rotation and allocation period per ward
4. Suggestions to improve clinical competence	4.1. Sufficient allocation period per ward 4.2. Need for adequate human and material resources 4.3. Effective communication including feedback from CSNs

CSNs, community service nurses.

### Theme 1: Facilitative experiences of community service nurses

Facilitative experiences of CSNs emerged as the first theme in this study. Below are the sub-themes with supporting verbatim statements from the participants who are referred to as ‘commserves’. The results are considered and discussed in relation to the literature.

#### Sub-theme 1.1. Improved clinical competence

A number of participants mentioned that community service is beneficial to them. To support this finding, one of the participants said:

‘…[*B*]ecause I was put into the deep end, I had to learn to swim, now I am a good swimmer in that ward, now they don’t want to lose me … [*laughs*].’ (FDG1, P4, female, 33 years old)

The results of this study concur with those of Govender et al. (2017) and Zaayman (2016) that community service programmes were perceived positively by participants as they provided them with the opportunity to gain knowledge and develop the requisite skills. These studies indicated that despite other challenges observed, CSNs felt confident and were able to adapt and work under pressure.

#### Sub-theme 1.2. Effective teamwork among staff members

Some of the participants felt that effective teamwork among staff members helped them as CSNs. For example, one of the participants said:

‘I think it depends on people that you are exposed to in the unit cause [*because*], in our shift, seriously, we are working very well; we collaborate very well with the staff members there.’ (FDG1, P5, male, 24 years old)

These results contrast with those of Parker et al. (2014) and Saghafi, Hardy and Hillege (2012). These studies reported that graduate nurses experienced stress from interactions with other nurses. Participants in these studies perceived other professional nurses as distant, unavailable and disinterested in helping them.

#### Sub-theme 1.3. Supportive nursing staff and other health professionals

From this study, it was evident that support received by CSNs from the nursing staff and doctors had a positive impact on their daily practice. This finding is supported by the following quotations: One participant said:

‘Even the assistant nurses, you just go to them and ask them …listen here help me with this or help me with that … I just had to go to the staff nurses and they showed me.’ (FDG2, P5, female, 27 years old)

To support this sub-theme, another participant said:

‘The doctors, in most wards, they are so nice. We’re actually supposed to learn from the sisters, but it’s fine doctors are teaching us.’ (FDG3, P5, female, 23 years old)

The study results concur with other studies conducted outside NWP. These studies include Roziers, Kyriacos and Ramugondo (2014) and Zaayman (2016) in the Western Cape province, and Govender et al. (2017) in KwaZulu-Natal. These studies revealed that despite the challenges experienced during placement of CSNs, the amount of support CSNs received assisted them in transitioning through the process of professional development.

#### Sub-theme 1.4. Constructive orientation and supervision

With regard to supervision and orientation, the majority of participants mentioned that they received orientation programmes that assisted them in the day-to-day routines of the wards. One of the participants noted:

‘At least they taught me how to do the doctor’s rounds, they orientated me very well, to do other stuff, what is it they expected from a commserve.’ (FDG3, P4, female, 26 years old)

This submission is in contrast with the results of the study by Kruse (2011) where lack of orientation and induction programmes prior to commencing duty was identified as a huge cause of anxiety and discord among CSNs.

### Theme 2: Defacilitative experiences of community service nurses

Defacilitative experiences of CSNs were the second theme that emerged from the findings of this study. Participants mentioned some negative experiences during their placement for community service. These experiences were perceived as having a negative impact on their ability to function and perform their tasks as expected by those who are supervising them.

#### Sub-theme 2.1. Unrealistic expectations

Participants reported that most of their managers and professional nurses displayed some unfair treatment and had unrealistic expectations. This might be attributed to the unavailability of a job description and scope of practice for this group of nurses as mentioned in the existing literature. To support this experience of unrealistic expectations, one of the participants mentioned:

‘I was the only sister, for the whole weekend, for 36 patients. It was expected of me to run the ward. I called the matron and I said to her I can’t do this, I’m a commserve.’ (FDG3, P5, female, 23 years old)

In a different study, Wilkes (2009) argues and recommends that supervisor and supervisee’s roles and responsibilities should be defined at the beginning of the placement to give realistic expectations and minimise misunderstandings and mistrust.

#### Sub-theme 2.2. Incompetent to perform basic nursing procedures

The majority of participants raised concerns regarding their incompetence with basic nursing procedures. Some of the participants said:

‘There’s an intercostal drain, and I don’t even know how to handle this patient with an IC drain.’ (FDG1, P4, female, 33 years old)

‘Sometimes I ask myself what if this patient reacts during blood transfusion, I don’t know what to do, who to call.’ (FDG2, P5, female, 27 years old)

According to Brown and Crookes (2016), nurses’ competence levels directly affected their ability to provide precise care for their patients. This study found that the majority of participants felt that they were incompetent in basic nursing procedures, which made it difficult for them to render quality care for the patients. This observation concurs with those in the study conducted by Shezi (2014), which indicated that not all CSNs were fully competent and independent to practice autonomously during their community service.

#### Sub-theme 2.3. Undesirable attitudes from some permanently employed staff

Some participants mentioned that they have experienced instances of negative attitudes from some permanently employed staff members, especially the professional nurses. One participant said:

‘But there’s a bad attitude … coming from the permanent employees who were here before you; they are having this attitude like … they don’t want working with commserves.’ (FDG3, P1, male, 29 years old)

According to Chaiklin (2011), attitudes are defined as a mental positioning with regard to a fact or state or a feeling or emotion towards an object. These results concur with studies conducted by Khunou (2016), Makua (2016) and Tsotetsi (2012), revealing that CSNs experienced some negative attitudes and feelings of unacceptance from their seniors during community service.

#### Sub-theme 2.4. Lack of interest in specific wards or departments (specifically maternity)

Some of the participants mentioned that they would prefer to be allocated to departments or wards that are of interest to them to work after completion of their community service. These participants mentioned that being allocated to a ward where one does not have an interest generates a negative attitude and lays blemish on their competence. Some participants said:

‘I’m not competent with maternity. It is just that, I’m not competent with maternity.’ (FDG3, P3, female, 30 years old)

‘I agree with you absolutely because I am so depressed working in maternity.’ (FDG3, P5, female, 23 years old)

In the study by Ross and Clifford (2002), participants suggested specific changes that could potentially be useful to nurse educationists regarding the selection of clinical placement during the training of nurses. These authors articulated that being able to choose their final placement area of specialisation would have been particularly beneficial, especially if this was an area they wanted to work in once qualified. In the case of this study, CSNs had no choice but to work wherever were allocated. Despite the good intentions of allocating CSNs to different wards for the benefit of being exposed to different clinical experiences, some participants felt it was unjust for them to be allocated to areas where they have no interest at all, specifically the maternity ward.

### Theme 3: Challenges faced by community service nurses during placement

All participants were faced with challenges that are not new to the specific cohort of CSNs in this study. These challenges were mentioned by participants in several studies conducted at the different provinces of the RSA.

#### Sub-theme 3.1. Shortage of human and material resources

Many of the participants from the three FGDs mentioned shortage of human and material resources as a major challenge influencing negatively on their competence. One participant said:

‘Like you have to do what you have to do with what you have, and doing a dressing without a sterile pack, it’s not correct, but you have to do it because that dressing has to be done.’ (FDG1, P1, female, 22 years old)

These study results replicate those of Thopola, Kgole and Mamogobo (2013), showing that participants perceived shortage of human and material resources as their greatest challenge during community service, which had a negative impact on the provision of quality nursing care.

#### Sub-theme 3.2. Unavailability of a job description or scope of practice

All participants highlighted the confusion caused by unavailability of a job description or scope of practice. This was perceived as a challenge, as it was difficult for CSNs to know exactly what is expected of them in undertaking their daily duties. One participant mentioned:

‘… And, because you don’t have any scope of practice, but you are a nurse. Whether the scope of practice, whether there’s something written, but you are a nurse.’ (FDG1, P4, female, 33 years old)

The results of this study confirm that of Govender et al. (2017). The results of these researchers revealed that CSNs find themselves holding ‘double’ titles, depending on the situation at their respective facilities, where at times CSNs were considered as being ‘students’, while on the other hand they were considered as replacements for registered nurse shortages (Govender et al. 2017). It is evident that the hospitals should work closely with their human resource departments to develop specific job descriptions for CSNs to minimise this confusion.

#### Sub-theme 3.3. Inconsistent rotation and allocation period per ward

Most of the participants in all FGDs displayed feelings of dissatisfaction owing to insufficient allocation period per discipline or inconsistent rotations to different wards or disciplines. This was identified as a disadvantage because this negatively affected the seasoning of clinical competence among participants. Some participants said:

‘So, you are competent now for 6 months in one ward, and now you are PN next year, and I know nothing about theatre. I’m not competent, never got the experience in my commserve.’ (FDG1, P3, female)

Adequate and logical rotation during placement provides opportunities to experience different wards or disciplines. The study conducted by Aggar et al. (2018) in Australia revealed that graduate nurses reported their preference for rotation at the commencement of their transition programme. The study also reported that graduates rated clinical rotations as very important during the 12-month transition period (Aggar et al. 2018).

### Theme 4: Suggestions for improving clinical competence

Some participants suggested that several issues should be considered to improve the comprehensive clinical competence of CSNs. Below are the sub-themes and verbatim quotations from participants.

#### Sub-theme 4.1. Sufficient allocation per ward

Some participants suggested that the allocation period per ward must be comprehensive enough to allow CSNs exposure to most of the wards during placement. One participant said:

‘So, I think they should at least maybe give two/two for the whole year so that you can get enough experience and then decide at the end of the year where do you want to work.’ (FDG3, P4, female, 26 years old)

These findings concur with those of Ndaba (2013) in Tshwane District where participants felt they were allocated to the busiest areas for most of the duration of the community service. They felt they were disadvantaged in their professional development because their allocation was limited only to particular disciplines like medical and surgical rather than the essential exposure to a variety of other disciplines such as maternity, geriatrics and paediatrics.

#### Sub-theme 4.2. Need for adequate human and material resources

Participants also suggested that the provision of adequate human and material resources ought to be considered if the comprehensive objectives of community service should be achieved. One participant said:

‘More staff are needed in the ward so that, if the other sister is busy with the rounds, the other sister must be busy with you on medication, to help you.’ (FDG3, P2, male, 28 years old)

The results of this study supported the recommendation by Thopola et al. (2013), that the Department of Health should address challenges of shortage of staff and material resources in order to improve the quality of nursing and midwifery care.

#### Sub-theme 4.3. Effective communication, including feedback from community service nurses

Participants also suggested that there should be effective communication that includes regular ward meetings and feedback as a strategy to improve clinical competence, which would lead to achieving the objectives of community service. Some participants said:

‘I do think they should get feedback from us before the end of the year, so that they know what we are experiencing so that they can correct such if there are any things that need to be corrected.’ (FDG3, P4, female, 26 years)

These results support the statement from Goodwin-Esola, Deelay and Powell (2009) who noted that constructive feedback during the transition period creates awareness of one’s ability in different areas and enables the novice to succeed in role transition. Similarly, Saghafi et al. (2012) showed that graduate nurses reported feedback as important in boosting their confidence. Furthermore, Marks-Maran et al. (2013) found that feedback enhances learning and is essential to support graduate nurses in identifying how their performance is perceived.

## Limitations

This study focussed on only three selected hospitals in the NWP. This means that results of this study cannot be generalised to other hospitals in the province or the country. However, similar studies could be conducted in other hospitals and primary health care settings where CSNs are allocated for their clinical service and in other provinces of the country in order to compare the results from different settings. However, study results could be applied in other provinces.

### Recommendations derived from the positive experiences of community service nurses

The study revealed that CSNs have facilitative experiences during their placement. It is recommended that facilities continue to improve on facilitative clinical competence experiences of CSNs by developing and implementing contextual transition programmes to orientate, supervise and support CSNs as well as encouraging peer collaborative learning and effective teamwork among staff members, including the CSNs.

### Recommendations derived from challenges encountered during placement

It is necessary for hospitals to develop proper orientation programmes and have mentors for CSNs as definitive strategies to provide support and improve clinical competence. It is also important for Professional Nurses (PNs) to set realistic expectations when they have allocated CSNs in their departments. Rotation or allocation per ward is important as this provides varied exposure for CSNs, with opportunities to experience different wards. Employment of adequate personnel and availability of human and material resources ensure the effectiveness of community service and as such should be integrated in the community service profile for CSNs. It is recommended that there must be a definitive job description and scope of practice for CSNs from the SANC that would inform decisions on delegation of duties for CSNs, thereby preventing the current role confusion and uncertainties with respect to their supervision.

### Recommendations for improved clinical competence

It is recommended that, for the clinical competence of CSNs to improve, CSNs need to be allocated adequate periods of clinical experience in each major clinical setting, with effective communication among all stakeholders. In tandem, hospital management needs to ensure adequate human and material resources. It is also imperative that there is a continuous professional development programme in place for CSNs during their placement to reinforce and refine their clinical competence, particularly in basic nursing procedures.

## Conclusion

Clinical competence of CSNs could be improved through effective collaboration among all stakeholders, including the hospital management, professional nurses, CSNs and the SANC. From this study, despite the positive and facilitative experiences, CSNs had defacilitative experiences and challenges that have a negative impact on their development and improvement of clinical competence. Many of the challenges reported by participants are consistent with those reported in the literature. There are various programmes or strategies in place to ensure that the new graduate nurses enter the working environment well prepared and emerge as competent practitioners. However, this study indicates that most of the challenges raised are the result of a shortage of human and material resources that impedes the effectiveness of community service. Suggestions were made in the hope that they would be considered to improve the clinical competence of CSNs. The results of this qualitative study contribute to the body of knowledge regarding clinical competence of CSNs during their placement.

## References

[CIT0001] Aggar C., Gordon C.J., Thomas T.H.T., Wadsworth L. & Bloomfield J, 2018, ‘Evaluation of a community transition to professional practice programme for graduate registered nurses in Australia’, *Nurse Education in Practice* 32, 101–107. 10.1016/j.nepr.2018.03.005 29605681

[CIT0002] Andre K. & Barnes L, 2010, ‘Creating a 21st century nursing work force: Designing a Bachelor of Nursing programme in response to the health reform agenda’, *Nurse Education Today* 30(3), 258–263. 10.1016/j.nedt.2009.09.011 19942327

[CIT0003] Babbie E, 2015, *The practice of social research*, 14th edn., Wadsworth/Thompson, Belmont, CA.

[CIT0004] Benner P, 2001, *From novice to expert: Excellence and power in clinical nursing practice*, Commemorative edn., Prentice-Hall, Upper Saddle River, New Jersey.

[CIT0005] Brown R.A. & Crookes P.A, 2016, ‘What are the “necessary” skills for a newly graduating RN? Results of an Australian survey’, *BMC Nursing* 15, 23 10.1186/s12912-016-0144-8 27051351 PMC4820976

[CIT0006] Chaiklin H, 2011, ‘Attitudes, behaviour and social practice’, *The Journal of Sociology & Social Welfare* 38(1), 31–54.

[CIT0007] Creswell J.W, 2014, *Research design: Qualitative, quantitative and mixed methods approaches*, 5th edn., Sage, Los Angeles, CA.

[CIT0008] Creswell J.W. & Creswell J.D, 2018, *Research design: Qualitative, quantitative and mixed methods approaches*, 5th edn., Sage, Los Angeles, CA.

[CIT0009] De Vos A.S., Strydom H., Fouché C.B. & Delport C.S.L, 2011, *Research at grass roots: For the social sciences and human service professions*, 4th edn., Van Schaik, Pretoria.

[CIT0010] Duchscher J.B, 2009, ‘Transition shock: The initial stage of role adaptation for newly graduated registered nurses’, *Journal of Advanced Nursing* 65(5), 1103–1113. 10.1111/j.1365-2648.2008.04898.x 19183235

[CIT0011] Goodwin-Esola M., Deely M. & Powell N, 2009, ‘Progress meeting: Facilitating role transition of new graduate’, *Journal of Continuing Education in Nursing* 40(9), 411–415. 10.3928/00220124-20090824-04 19754028

[CIT0012] Govender S., Brysiewicz P. & Bhengu B, 2017, ‘Pre-licensing experiences of nurses performing compulsory community service in KwaZulu-Natal, South Africa: A qualitative study’, *Journal of Africa Nursing Sciences* 6, 14–21. 10.1016/j.ijans.2017.01.001

[CIT0013] Hansen-Salie N. & Martin P.D, 2014, ‘The perceptions and factors influencing the competency in newly qualified professional nurses working in private hospitals in the Western Cape, South Africa’, *African Journal for Physical, Health Education, Recreation and Dance* October (Suppl. 1:2), 538–553.

[CIT0014] Institute of Medicine (IOM), 2011, *The future of nursing: Leading change, advancing health*, The National Academic Press, Washington, DC.

[CIT0015] Khunou S.H, 2016, ‘Development of a mentoring programme for community service nurses in the North-West province public health facilities’, Unpublished thesis, North-West University Mafikeng Campus, Mmabatho.

[CIT0016] Kruse B, 2011, ‘Retaining community service nurses in the Western Cape public health sector’, Master’s dissertation, University of Stellenbosch, Cape Town.

[CIT0017] Kubin L. & Fogg N, 2010, ‘Back-to-basics boot camp: An innovative approach to competency assessment’, *Journal of Paediatric Nursing* 25(1), 28–32. https://doi.org/10.1016.j.pedn.2008.07.004 10.1016/j.pedn.2008.07.004 20117673

[CIT0018] Makhakhe A.M, 2010, ‘Nurses’ experiences of the transition from student to professional practitioner in a public hospital in Lesotho’, Professional Nursing Science dissertation, Potchefstroom Campus of the North-West University, North-West University, Potchefstroom.

[CIT0019] Makua M.G, 2016, *Induction and professional development support of newly qualified professional nurses during community service*, University of South Africa, Pretoria, viewed 23 November 2018, from http://hdl.handle.net/10500/22273.

[CIT0020] Marks-Maran D., Ooms A., Tapping J., Muir J., Phillips S. & Burke L, 2013, ‘A preceptorship programme for newly qualified nurses: A study of preceptees’ perceptions’, *Nurse Education Today* 33(11), 1428–1434. 10.1016/j.nedt.2012.11.013 23260624

[CIT0021] Ndaba B, 2013, ‘Lived experiences of newly qualified professional nurses doing community service in Midwifery section in one Gauteng hospital’, Health Studies dissertation, University of South Africa, Pretoria.

[CIT0022] Netshisaulu K.G. & Maputle M.S, 2018, ‘Expected clinical competence from midwifery graduates during community service placement in Limpopo province, South Africa’, *Health SA Gesondheid* 23(0), a1166 10.4102/hsag.v23i0.1166 PMC6917449 31934392

[CIT0023] Parker V., Giles M., Lantry G. & McMillan M, 2014, ‘New graduate nurses’ experiences in their first year of practice’, *Nurse Education Today* 34(1), 150–156. 10.1016/j.nedt.2012.07.003 22857819

[CIT0024] Pienaar A.J, 2017, ‘Learning and asserting an African indigenous health research framework’, in Ngulube P. (ed.), *Handbook of research on theoretical perspectives on indigenous knowledge systems in developing countries*, pp. 85–99, Hersley, Pennsylvania, USA.

[CIT0025] Polit D.F. & Beck C.T, 2012, *Nursing research: Generating and assessing evidence for nursing practice*, 9th edn., Lippincott Williams & Wilkins, London.

[CIT0026] Riddle D., Baker K. & Sapp A, 2016, ‘Evaluation of testing as a method to assess continued competency in nurse anaesthesia practice: A systematic review’, *AANA Journal* 84(4), 239–245. 30501149

[CIT0027] Ross H. & Clifford K, 2002, ‘Research as a catalyst for change: The transition from student to registered nurse’, *Journal of Clinical Nursing* 11(4), 545–553. 10.1046/j.1365-2702.2002.00610.x 12100651

[CIT0028] Roziers R.L., Kyriacos U. & Ramugondo E.L, 2014, ‘Newly qualified South African nurses’ lived experience of the transition from student to community service nurse: A phenomenological study’, *Journal of Continuing Education in Nursing* 45(2), 91–100. 10.3928/00220124-20140122-01 24443805

[CIT0029] Saghafi F., Hardy J. & Hillege S, 2012, ‘New graduate nurses’ experiences of interactions in the critical care unit’, *Contemporary Nurse* 42(1), 20–27. 10.5172/conu.2012.42.1.20 23050568

[CIT0030] Shezi B.E, 2014, ‘The needs of community service nurses with regard to supervision and clinical accompaniment’, Master’s dissertation, North-West University, Potchefstroom.

[CIT0031] Snell L.A. & Daniels F.M, 2014, ‘Perceptions of professional nurses regarding clinical competence of community service practitioners from degree and diploma programmes offered in the Western Cape’, *African Journal for Physical, Health Education, Recreation and Dance (AJPHERD) Supplement* 1(1), 142–153.

[CIT0032] South Africa Department of Health, 2006, *Health on community service by health professionals*, Department of Health, Pretoria, viewed 25 June 2019, from https://www.gov.za/health-community-service-health-professionals.

[CIT0033] South Africa Nursing Council (SANC), 2005, *Nursing Act, Act no. 33 of 2005*, Government Printer, Pretoria.

[CIT0034] South African Nursing Council (SANC), 2007, *Regulations relating to performance of community service*, Regulation R765, in terms of the Nursing Act, 2005 (Act 33 of 2005, as amended), Government Printer, Pretoria.

[CIT0035] Theisen J.L. & Sandau K.E, 2013, ‘Competency of new graduate nurses: A review of their weaknesses and strategies for success’, *Journal of Continuing Education in Nursing* 44(9), 406–414. 10.3928/00220124-20130617-38 23799789

[CIT0036] Thopola M.K., Kgole J.C. & Mamogobo P.M, 2013, ‘Experiences of newly qualified nurses at University of Limpopo, Turfloop Campus executing community service in Limpopo Province, South Africa’, *African Journal for Physical, Health Education, Recreation and Dance* March 2013 (Supplement 1), 161–181.

[CIT0037] Tsotetsi A.D, 2012, ‘Experiences and support of the newly qualified four-year trained professional nurses placed for remunerated community service in Gauteng Province’, Master’s dissertation, University of Pretoria, Pretoria.

[CIT0038] Wilkes Z, 2009, ‘The student-mentor relationship: A review of the literature’, *Nursing Standard* 20(37), 42–47. 10.7748/ns.20.37.42.s55 16764399

[CIT0039] Zaayman E.S, 2016, ‘Professional nurses’ experiences of their community service placement at a secondary academic hospital in the Western Cape’, Master’s dissertation, University of the Western Cape, Bellville, Western Cape.

